# Rumen Fermentation Characteristics in Pre- and Post-Weaning Calves upon Feeding with Mulberry Leaf Flavonoids and *Candida tropicalis* Individually or in Combination as a Supplement

**DOI:** 10.3390/ani9110990

**Published:** 2019-11-18

**Authors:** Luxin Kong, Chuntao Yang, Lifeng Dong, Qiyu Diao, Bingwen Si, Junnan Ma, Yan Tu

**Affiliations:** Feed Research Institute, Chinese Academy of Agricultural Sciences, Key Laboratory of Feed Biotechnology of the Ministry of Agriculture and Rural Affairs, Beijing Key Laboratory for Dairy Cow Nutrition, Beijing 100081, China; kongluxin@caas.cn (L.K.); yangchuntao0808@163.com (C.Y.); donglifeng@caas.cn (L.D.); diaoqiyu@caas.cn (Q.D.); sibingwen@caas.cn (B.S.); manan014@163.com (J.M.)

**Keywords:** *Candida tropicalis*, growth performance, mulberry leaf flavonoid, rumen fermentation characteristics

## Abstract

**Simple Summary:**

Supplementing diets with yeast or yeast cultures is recognized as a common practice in modern dairy farming because of the positive effects of yeast on production, health, and immunity performance in ruminants. Recent studies have demonstrated that supplementation with *Candida tropicalis* has potential benefits by improving fibrous material digestion and antioxidant function, and enhancing the microbial activities in the rumen. Meanwhile, flavonoids, as secondary plant metabolites, are ubiquitously present in plants used for livestock feed, have health-promoting properties, including antioxidative, anti-inflammatory, and metabolic effects. However, in practice, supplementation with monostrain probiotics or individual additives has not enhanced production as expected. Therefore, we hypothesize that multispecies probiotics or the combination of yeast and phytochemicals could be compatible with each other and act synergistically. The results showed that supplementation with *C. tropicalis* or flavonoids improved rumen fermentation, but supplementation with *C. tropicalis* had limited effects on increasing growth performance and decreasing fecal scores compared with flavonoid supplementation. However, the combination of *C. tropicalis* and flavonoids did not show a synergistic effect on health or rumen fermentation compared with use of flavonoids alone in pre- and post-weaning calves.

**Abstract:**

Although flavonoids or yeast have been used as feed additives to improve the production efficiency and health of adult cattle, little information is available on their effects on rumen fermentation in calves. The objective of this study was to investigate the effects of feed supplementation with mulberry leaf flavonoids and *Candida tropicalis* on performance, blood parameters, and rumen fermentation characteristics during pre-weaning and post-weaning periods. Forty-eight Holstein calves were used in a four-groups trial and were supplemented with (1) no yeast or flavonoids (CON), (2) active dry yeast (ADY; *C. tropicalis*, 5.0 × 10^9^ CFU/d), (3) flavonoids (FLA; 3 g/d), or (4) yeast and flavonoids (YF; *C. tropicalis*, 5.0 × 10^9^ CFU/d; flavonoids, 3 g/d). The feeding strategy was as follows: milk replacer was offered at 12% of body weight in two meals per calf each day at age 21 d, and a gradual weaning process was adopted at age 50 to 56 days. Data of daily feed intake, body weight, and serum and rumen fermentation parameters were obtained at 28, 42, 56, and 80 d ages, respectively. A significant time effect and interaction between treatment and time was found for average daily gain, feed efficiency, total volatile fatty acid concentration, and proportion of propionate in calves (*p* < 0.05). Average daily gain and feed efficiency increased during post-weaning and overall periods for calves in FLA and YF groups compared with CON and ADY groups (*p* < 0.05). A reduction of fecal scores with supplementation was found in FLA and YF groups (*p* < 0.05). Rumen fluid pH and ammonia nitrogen concentration remained constant across the groups, whereas total volatile fatty acid concentration and molar proportion of propionate significantly increased during the pre-weaning and overall periods in FLA and YF groups (*p* < 0.05). Calves in YF group had the highest serum concentrations of IgG and IgA during the overall period (*p* < 0.05). Additionally, serum β-hydroxybutyric acid concentration was higher in ADY and FLA groups during the post-weaning period (*p* < 0.05). Supplementation with *C. tropicalis* showed little effect on increasing growth performance and health compared with flavonoids alone. Meanwhile, the combination of *C. tropicalis* and flavonoids was not synergistic with respect to improving health and rumen fermentation compared with use of flavonoids alone in pre- and post-weaning calves (*p* > 0.05).

## 1. Introduction

Incorporation of yeast and yeast cultures into the diets of ruminants is recognized as a common practice in modern dairy farming because yeast has positive effects on production, health, and immunity performance [1]. Unlike *Saccharomyces cerevisiae*, *Candida tropicalis* is an opportunistic pathogen that causes disease [2,3]. However, publications have reported that *C. tropicalis* is commonly detected in the gastrointestinal tract of healthy humans [4,5] and has no harmful effects on animal health [6]. Furthermore, recent studies demonstrated that supplementation with *C. tropicalis* has potential benefits by improving fibrous material digestion and antioxidant function, and enhancing the microbial activities in the rumen [7,8]. Moreover, some studies have shown that supplementation with monostrain probiotics does not positively influence animals because such probiotics have little chance to colonize the gastrointestinal tract [9] or control multifactorial diseases [10]. Timmerman et al. [9] observed that strains used in multistrain, multispecies probiotics or the combination of yeast and phytochemicals are compatible with each other and act synergistically.

Phytochemicals may have potential health benefits for animals and can serve as alternatives to pharmaceuticals [11,12]. Mulberry leaves have been traditionally considered as an alternative high-quality feed for farm animals in China, owing to their high nutritive value as well as their flavonoid contents [13]. Recent studies have demonstrated that mulberry-derived flavonoids have the potential to enhance or stabilize animal performance and health [14,15]. Flavonoids are secondary plant metabolites and are ubiquitously present in plants used for livestock feed [16]. Many plant-derived extracts, which contain considerable amounts of various flavonoids, have health-promoting properties (e.g., antioxidative, anti-inflammatory, and metabolic effects), as well as other functional characteristics, such as modulating the expression and activities of several enzymes involved in lipid and carbohydrate metabolism [17,18]. Therefore, we hypothesized that the combined use of *C. tropicalis* and flavonoids would have greater effects on the growth performance, health, and rumen fermentation characteristics of pre- and post-weaning calves compared with the use of either supplement alone.

## 2. Materials and Methods

### 2.1. Flavonoids and Active Dry Yeast

The natural flavonoids extracted from Mulberry (*Morus alba*) leaves were obtained from Xi’an Feida Bio-Tech (Shanxi, China). The main components (wt/wt) were flavones (65.0%), flavonols (20%), and other polyphenols (15.0%) that share a common flavan core (15 carbon atoms). Active dry yeast (*C. tropicalis*, 5.0 × 10^9^ CFU/g) was purchased from Huanong Biological Engineering (Beijing, China). Before commencement of animal trials, the number of viable cells in 1 g of dehydrated active dry yeast was determined by plating cells on yeast extract peptone dextrose agar at 37 °C. The active dry yeasts were kept at 4 °C, and viable cells were examined weekly to ensure their stability.

### 2.2. Animals, Diets, and Experimental Design

This study was conducted at a commercial dairy farm (Sanyuan Co., Beijing, China) in compliance with the animal protection regulations approved by the Animal Ethics Committee of the Chinese Academy of Agricultural Sciences (with protocol FRI-CAAS-20140812).

A total of 48 Chinese Holstein bull calves with mean body weight of (BW) 46 ± 5.9 kg were randomly allocated to one of four experimental groups from age 21 to 80 d. All calves were fed the same diet before age 28 d. Each group had 12 replicates, with 1 calf per replicate. Calves from the four groups at age 28 d were supplemented with (1) no yeast or flavonoids (CON), (2) *C. tropicalis* (ADY; 1 g/calf per day), (3) flavonoids (FLA; 3 g/calf per day), or (4) *C. tropicalis* and flavonoids (YF; *C. tropicalis*, 5.0 × 10^9^ CFU/d; flavonoids, 3 g/d). Milk replacer (12% BW per calf per day; Table 1) was offered twice daily at 08:00 and 16:00 before weaning. The weaning process commenced at age 50 d and milk replacer was gradually replaced with starter until age 56 d. The flavonoids dose was calculated based on our previous results [13] and was equivalent to 65 mg of flavonoids/kg of BW. The dose of active dry yeast used in the present study was calculated based on the results in dairy cows observed by Chung et al. [19] and Jing et al. [20]. During the pre-weaning period, the supplement was individually mixed with milk replacer liquid and fed directly to the calves in the morning feeding. During the post-weaning period, the supplement was individually hand-mixed with 100 g of starter to ensure that all the supplement had been consumed, and then more starters were subsequently fed to individual calves. The ingredients of the starter (as % of dry matter, DM) were corn (20%), extruded corn (22.9%), soybean meal (20%), extruded soybean (18%), dried whey (5%), wheat bran (10%), calcium hydrogen phosphate (0.8%), limestone (1.8%), salt (0.5%), and premix (1%). The compositions of premix (per kilogram of starter) were vitamin A (15,000 IU), vitamin D (5000 IU), vitamin E (50 mg), Fe (90 mg), Cu (12.5 mg), Mn (30 mg), Zn (90 mg), Se (0.3 mg), I (1.0 mg), and Co (0.5 mg). The milk replacer (patent for invention, CN 02128844.5) was provided by the Beijing Precision Animal Nutrition Research Center (Beijing, China). The chemical compositions of starter and milk replacer are showed in Table 1. Clean, fresh water and dry pelleted starter were provided ad libitum throughout the trial. Calves were housed in individual pens (3.0 m × 1.2 m, 3.6 m^2^/animal) surrounded by fencing (1.1 m high). Two metal supports were attached to the side of the fence to support feeders.

### 2.3. Measurements, Sample Collection, and Analysis

Throughout the experiment, the intakes of milk replacer and starters for each calf were recorded daily, and representative feed samples were collected weekly and frozen at −20 °C for subsequent analyses. The BW of each calf was recorded at ages 28, 42, 56, and 80 d to calculate average daily gain (ADG) and feed efficiency. Fecal consistency was scored daily during the morning milk feeding using a 1 to 4 scale according to Heinrichs et al. [21]. Fecal score >2 for 3 consecutive days was considered to reflect diarrhea. In this trial, only a subset of calves (n = 7 per group) with BW similar to the group average were considered for blood variable and rumen fermentation parameter analyses. Blood samples were taken by jugular venipuncture at ages 28, 42, 56, and 80 d before the morning feeding. Afterward, serum was recovered by centrifugation for analysis of blood metabolite parameters. The concentrations of IgG, IgA, IgM, and epidermal growth factor (EGF) were determined by ELISA using cow IgA, IgG, IgM, and EGF kits from Bethyl Laboratories (Montgomery, TX, USA). The β-hydroxybutyric acid (BHBA) concentration was measured using a BHBA dehydrogenase reagent kit (310-UV, Sigma, St. Louis, MO, USA). Rumen fluid was collected at ages of 28, 42, 56, and 80 d using a copper probe (length 5 cm, internal diameter 1 cm) attached to a plastic tube, protected by stainless steel springs and designed specifically for calves. The probe was inserted into the mouth of the calf and fed down the rumen. A 200-mL syringe was used to withdraw rumen fluid. The first 50 mL of fluid was discarded, and subsequent fluid was collected. Rumen fluid pH was immediately determined using a handheld pH meter that was calibrated before each reading (Testo 205, Testo AG, Lenzkirch, Germany). Rumen fluid samples were strained through four layers of cheesecloth to remove large feed particles. Samples for ammonia N concentration (NH_3_-N) analyses were preserved by mixing 5 mL filtered rumen fluid with 5 mL of 0.2 M HCl; samples for analysis of volatile fatty acid (VFA) concentrations were prepared by mixing 5 mL filtered rumen fluid with 1 mL of 0.5 M H_2_SO_4_. The concentration of NH_3_-N was determined using the methods described by Dong et al. [22]. Individual and total VFA concentrations in aliquots of rumen fluid were determined with a flame ionization detector in a gas chromatograph (GC522, Wufeng Instruments, Shanghai, China), using a 15-mL semi-capillary glass column (0.53 mm in diameter) packed with Chromosorb 101 (Johns-Monville, Denver, CO, USA), with N_2_ as carrier gas at a column temperature of 120 °C [23].

### 2.4. Statistical Analysis

Data was analyzed separately for the pre-weaning period (age 28 to 50 d), post-weaning period (age 56 to 80 d), and overall period (age 28 to 80 d). Data for growth performance, serum, and rumen fermentation were analyzed as a randomized complete design. Fecal scores were pooled by week for analysis. A repeated-measure ANOVA was conducted using the Mixed procedure in SAS (Statistical Analysis System) software version 9.2 (SAS Institute Inc., Cary, NC, USA), based on the statistical model:

Y_ijk_ = µ + T_i_ + D_j_ + (TD)_ij_ + C_(i)k_ + e_(ij)k_ (1)

where Y_ijk_ = dependent variable, µ = overall mean, T_i_ = fixed effect of treatment (I = 1, 2, 3, 4), D_j_ = fixed effect of day (j = 28, 42, 56, 80), (TD)_ij_ = fixed interaction of treatment and day, C_(i)k_ = random effect of each calf within the group, and e_(ij)k_ = residual error. 

The AR (Autoregressive model) (1) covariance structures were used based on model fit, day (week) was the repeated variable, and calf by treatment was the subject. Denominator degrees of freedom were adjusted by the Kenward–Rogers method. Significance was declared at *p* < 0.05 and the comparison of the means was carried out with the LSD test.

## 3. Results

### 3.1. Intake, Growth Performance, and Fecal Score

Throughout the study, the effect of treatment or treatment × time was not observed for feed DM intake (milk replacer or starter) (*p* > 0.05, Table 2). However, a significant difference was detected among groups for ADG and feed efficiency (daily gain/daily DM intake, kg/kg) during post-weaning and overall periods (*p* < 0.05). The ADG of calves was greater in FLA and YF groups compared with CON and ADY groups during the post-weaning period (0.77 and 0.79 vs. 0.69 and 0.70 kg/d; *p* < 0.05) and overall period (0.63 and 0.64 vs. 0.58 and 0.60 kg/d; *p* < 0.05). During the post-weaning period, feed efficiency was higher in the ADY and FLA groups, followed by the YF and CON groups (*p* < 0.05), and it was greater in FLA group than that of YF and CON groups during the overall period (*p* < 0.05). Treatment and time affected fecal score (treatment, time, *p* < 0.05) during the pre-weaning, with lower (treatment, *p* = 0.039) score in FLA and YF groups compared with CON group.

### 3.2. Rumen Fermentation Characteristics

**Figure 1:**
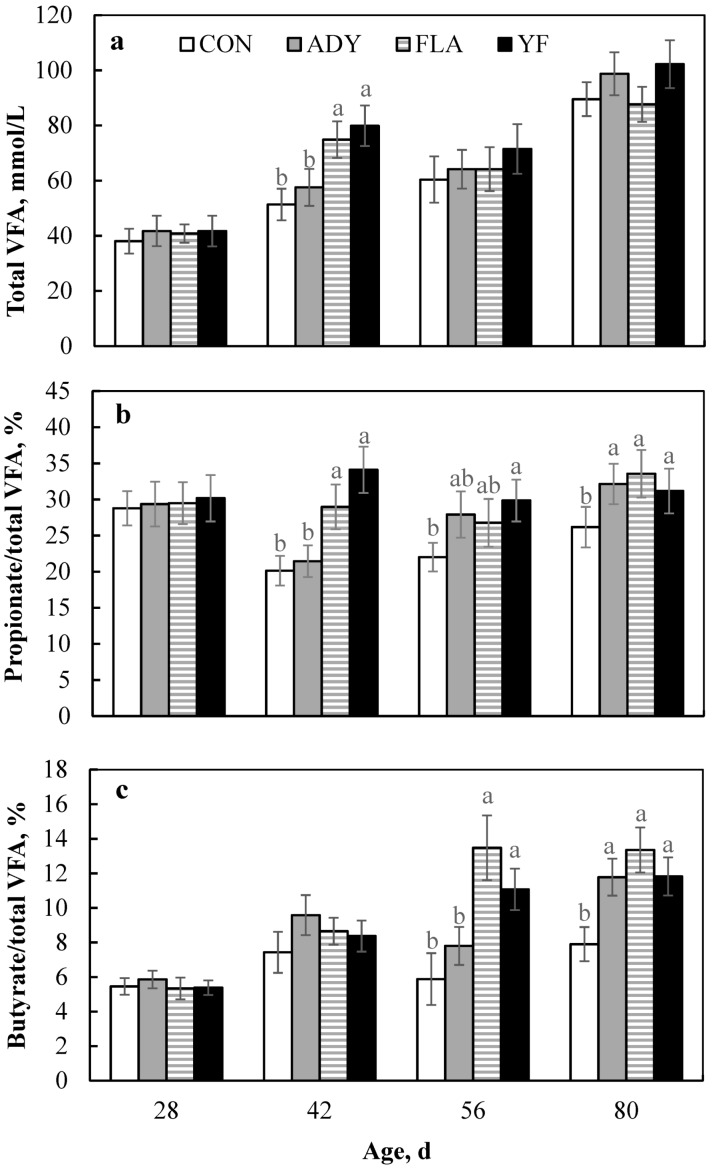
Effects of supplementation with *Candida tropicalis* and mulberry leaf flavonoids individually or in combination on total volatile fatty acid (VFA) (**a**) and molar proportions of propionate (**b**) and butyrate (**c**) in calves at ages 28, 42, 56, and 80 d. CON = no yeast or flavonoids fed; ADY = *Candida tropicalis* as supplement; FLA = mulberry leaf flavonoids as supplement; YF = combination of *Candida tropicalis* and mulberry leaf flavonoids as supplement. Within sets of bars, those marked with different letters differ significantly (*p* < 0.05).

Rumen fluid pH and NH_3_-N concentration across groups were similar during the pre-weaning, post-weaning, and overall periods (*p* > 0.05, Table 3). Total VFA concentrations in the YF group were higher (*p* < 0.05), especially at age 42 d (Figure 1a), compared with that in the CON and ADY groups during the pre-weaning and overall periods, whereas VFA concentration was similar among groups during the post-weaning period (*p* > 0.05). Calves in the YF group had a higher molar proportion of propionate (especially at age 42 d; Figure 1b) and lower acetate/propionate ratio compared with the CON group during the pre-weaning (*p* < 0.05) and overall (*p* < 0.05) periods. Additionally, calves fed yeast, flavonoids, or both have a higher molar proportion of butyrate at age 80 d (*p* < 0.05; Figure 1c), and this tendency was high compared with CON calves during the post-weaning period (*p* = 0.055).

### 3.3. Blood Biomarkers

**Figure 2:**
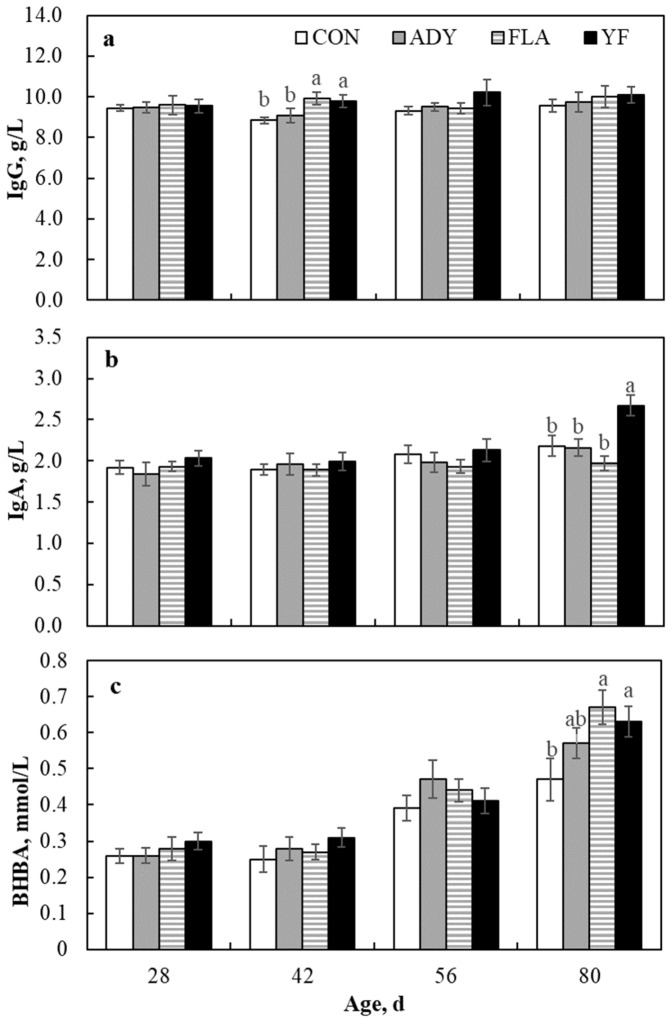
Effects of supplementation with *Candida tropicalis* and mulberry leaf flavonoids individually or in combination on serum concentrations of IgG (**a**), IgA (**b**), and β-hydroxybutyric acid (BHBA, (**c**)) in calves at ages 28, 42, 56, and 80 d. CON = no yeast or flavonoids fed; ADY = *Candida tropicalis* as supplement; FLA = mulberry leaf flavonoids as supplement; YF = combination of *Candida tropicalis* and mulberry leaf flavonoids as supplement. Within sets of bars, those marked with different letters differ significantly (*p* < 0.05).

A significant difference was observed for serum IgG and IgA concentrations during the pre-weaning, post-weaning, or overall periods (*p* < 0.05; Table 4). The IgG concentrations measured for the FLA and YF groups were higher, especially at age 42 d (Figure 2a), than those for the CON and ADY groups during the pre-weaning period (treatment, treatment × time, *p* < 0.05), and were higher compared with the CON group during the overall period (treatment, treatment × time, *p* < 0.05). Similar results were observed for IgA concentration, with significant differences observed between the YF group and other groups during the post-weaning (at age 80 d, Figure 2b) (treatment, treatment × time, *p* < 0.05) and overall periods (treatment, *p* = 0.031). Throughout the study period, however, the IgM concentration did not differ among groups (*p* > 0.05). The serum BHBA concentration increased with the age of calves (time, *p* < 0.05), and BHBA concentrations were higher in the ADY and FLA groups than other groups during the post-weaning period (treatment, treatment × time, *p* < 0.05), especially at age 80 d (Figure 2c). The result for EGF concentration in the CON group was not different compared with the other groups (*p* > 0.05), but there was a significant difference between the FLA and ADY groups during the pre-weaning period (treatment, *p* = 0.047).

## 4. Discussion

### 4.1. Growth and Health Performance 

Flavonoids have been widely investigated and increasingly used in monogastric species and ruminant production systems, and have improved growth performance, health, and rumen fermentation conditions [24]. Some previous studies have reported that yeast is an effective alternative to antibiotics in ruminants [25,26], but these trials reported little benefit to calf performance [27,28]. Similar to the results of our present study, oral administration of *C. tropicalis* did not improve DM intake or ADG of calves [28]. However, flavonoids or a mixture of flavonoids and yeast had beneficial effects on ADG and feed efficiency of calves during the post-weaning period, indicating that flavonoid supplementation may help alleviate stress during weaning [29]. Less stressful and earlier weaning improves calf health and the economic impact of raising calves. Compared with flavonoids alone, however, the combined use of *C. tropicalis* and flavonoids did not have a synergistic effect. Balcells et al. [24] found that feeding flavonoids had little effect on ADG (1.16 vs. 1.09 kg/d) and feed conversion ratio (6.80 vs. 7.28 kg/kg, *p* > 0.05) in adult heifers. This discrepancy may be attributed to the fact that the epithelium in newborn animals is comprised mainly of vacuolated epithelial cells [30,31], which absorb flavonoids or metabolites that retain a flavonol structure, and these cells are not present in older animals [32]. Flavonoids have structures similar to those of estrogenic hormones, and they share certain functional characteristics, such as modulation of expression and activity of key lipid and carbohydrate metabolic enzymes, which enhance anabolism [17,18]. Mechanistically, this may explain why flavonoid supplementation contributes to improved ADG and feed efficiency for younger animals, as we observed in the present study. Future work will be required to substantiate these findings in detail using larger sample sizes.

Although *C. tropicalis* was previously documented as an opportunistic pathogen [3], it is commonly detected in the gastrointestinal tract of healthy humans [4,5] and contributes to the digestion of fibrous material in production animals [6,7]. Magalhães et al. [1] reported a decrease in fecal scores when calves were fed *S. cerevisiae*. So far, however, the effects of *C. tropicalis* on health conditions have not been investigated in ruminants. In the present study, supplementing calves with *C. tropicalis* did not improve animal health compared with flavonoids. Flavonoids may decrease fecal scores in calves by disrupting pathogen cell membrane integrity and metabolism, thereby inhibiting pathogen growth. Oral administration of a mixture of yeast and flavonoids yielded the same effect compared with flavonoids alone, suggesting that feeding calves a combination of yeast and flavonoids does not have a synergistic effect.

### 4.2. Rumen Fermentation Parameters

We found that rumen pH was not significantly affected by supplementation with flavonoids or yeast or the combination of both during pre-weaning, post-weaning, and the overall period, which is consistent with the findings of Oskoueian et al. [33]. An *in vitro* study conducted by Seradj et al. [34] showed that flavonoids supplementation did not influence the pH of rumen fluid from steers. Bayat et al. [35] reported that supplementation with live yeast strains had little effect on rumen fluid pH, which was in agreement with the findings of Aikman et al. [36] showing that supplementation with *Megasphaera elsdenii* did not alter the rumen pH in early lactating dairy cows. Some research suggests that rumen pH is affected not only by differences in feed, saliva, and rate of passage in the rumen, but also by fermentation products (e.g., NH_3_-N, VFA, and lactic acid) [36]. In the present study, the concentrations of NH_3_-N and acetate were minimally affected throughout the experiment, which may contribute to the observed constant rumen pH and fermentation conditions. Moreover, the molar proportions of propionate and total VFA concentration were significant different across groups during certain specific periods, suggesting that yeast or flavonoids can be utilized as fermentable substrates. For flavonoids, some research suggests that they can be readily degraded by microbes, and their derivatives enhance rumen microbial activity in adult ruminants [14]. Increases in propionate concentration could be attributed to enhanced growth of propionate-producing bacteria in the rumen [14], and consequently decreased acetate/propionate ratio in rumen. Smith et al. [37] found phenolic acids and nonaromatic fermentation products in the rumen as the result of microbial degradation of flavonoids. Thus, these byproducts could play a role as alternative carbon sources for rumen microbes. On the other hand, diet supplement with flavonoids increases the butyrate concentration in the rumen, and butyrate is regarded as a signaling molecule that indirectly stimulates epithelial proliferation [38]. This suggests that flavonoids may contribute to the rumen development, but the mechanism remains unclear. However, rumen VFA responses to yeast supplementation are inconsistent [35,39]. For example, Kumprechtová et al. [27] found that addition of live yeast to the diet increased rumen VFA concentrations, whereas Bayat et al. [35] demonstrated no influence of yeast on rumen VFA concentrations for lactating cows fed grass silage diets. Little research has been published concerning the effect of *C. tropicalis* supplementation on rumen VFA production. In the current study, adding *C. tropicalis* alone to the diet did not increase VFA concentrations, but rather increased the propionate concentration, although the combination of yeast and flavonoids had greater effects. Marrero et al. [7] also established that *C. tropicalis* has a greater ability to digest soluble carbohydrates compared with *S. cerevisiae*, and thus *C. tropicalis* may convert more substrates to glucose, which is needed by younger animals.

### 4.3. Blood Parameters

In the current study, supplementation with mulberry leaf flavonoids alone or combined with *C. tropicalis* reduced the fecal scores. This coincided with increased concentrations of IgG, IgM, and IgA over the course of the study. It has been widely recognized that immunoglobulin concentration can be used as an indicator of calf health [40]. IgA has the potential to reduce pathogenic bacteria by combination with innate nonspecific defense mechanisms. IgM plays an important role at the initial stage of antibody response. In the present study, supplementation with a combination of yeast and flavonoids resulted in increased concentrations of serum IgG and IgA, suggesting that the combined supplementation may help stimulate the immune response more than either supplement alone. The mechanism by which phytogenic feed additives increase serum immunoglobulin concentrations is largely unknown. It is possible that bioactive molecules derived from flavonoids (e.g., flavone, flavonols, etc.) act as additional ligands for Fc receptors, which bind to IgG and stimulate the immune response [41]. Probiotics as immunomodulators interact with the intestinal resident microflora and epithelial and immune cells to stimulate immune function, leading to antibody production [42]. Higher levels of immunomodulators increase antibody production, which neutralizes antigen or pathogenic bacteria via a systemic immune response that reduces stress responses and increases the growth of younger animals. However, the mechanisms by which yeast and flavonoids interactively affect immune function remain poorly defined.

Butyrate is reportedly converted to BHBA in the rumen wall before appearing in the portal circulation [10]. Therefore, as the rumen wall is metabolically nonfunctional in newborn calves, the concentration of BHBA might be relatively low compared with that in adult ruminants. The concentration of BHBA in calves increased over time, and supplementation with yeast or flavonoids alone yielded the same result for the concentration of butyrate. This change suggests that rumen functional development is enhanced by supplementation with yeast and flavonoids, and that supplementation also improves the energy source from glucose change to VFA [43]. Serum EGF concentration was not affected by supplementation with flavonoids or yeast or a combination of both. Interestingly, calves fed flavonoids had higher serum EGF concentrations than those fed yeast during the pre-weaning period. This may be attributed to the molecular structure of flavonoids being similar to estradiol, which can regulate the expression of EGF and EGF receptors, and to *C. Tropicalis*, which in the gastrointestinal tract may influence the absorption of EGF.

## 5. Conclusions

Supplementation with *C. tropicalis* or flavonoids increased the proportion of propionate among rumen fermentation products, but supplementation with *C. tropicalis* had limited effects on increasing growth performance and decreasing fecal scores compared with flavonoids supplementation. Moreover, the combination of *C. tropicalis* and flavonoids did not have a synergistic effect on health or rumen fermentation compared with use of flavonoids alone in pre- and post-weaning calves. Additionally, a time effect or interaction between treatment and time was found for ADG, feed efficiency, total volatile fatty acid concentration, and proportion of propionate in calves. Further investigation is needed to illuminate the mechanism of flavonoid supplementation to promote rumen development and microbial composition in calves.

## Figures and Tables

**Table 1:** Chemical composition (% of dry matter basis) of starter and milk replacer used in the present study.

Item	Starter	Milk Replacer
Dry matter, % as fed	85.36	95.36
Organic matter	92.21	94.85
Crude protein	19.08	24.27
Gross energy, MJ/kg of DM	15.45	19.86
Neutral detergent fiber	18.59	4.02
Acid detergent fiber	10.65	2.11
Ether extract	2.21	12.85
Calcium	1.09	1.07
Phosphorus	0.47	0.48

**Table 2:** Effects of supplementation with *Candida tropicalis* and mulberry leaf flavonoids individually or in combination on growth performance and fecal scores in Holstein calves during pre-weaning, post-weaning, and overall periods.

Item ^1^	Treatment ^2^				SEM ^3^	*p*-Value		
	CON	ADY	FLA	YF		Treatment	Time	Treatment × Time
DM intake, kg/d								
Milk replacer								
Pre-weaning	0.76	0.76	0.78	0.77	0.02	0.618	0.445	0.592
Starter								
Pre-weaning	0.25	0.27	0.27	0.30	0.02	0.221	0.194	0.217
Post-weaning	1.40	1.49	1.51	1.65	0.03	0.130	0.691	0.541
Overall	0.96	0.99	1.02	1.11	0.02	0.205	0.427	0.313
Total DMI, kg/d								
Pre-weaning	1.00	1.03	1.05	1.08	0.02	0.124	0.242	0.121
Overall	1.12	1.13	1.16	1.22	0.02	0.212	0.117	0.422
Average daily gain, kg/d								
Pre-weaning	0.48	0.51	0.48	0.50	0.03	0.134	0.281	0.226
Post-weaning	0.69 ^b^	0.70 ^b^	0.77 ^a^	0.79 ^a^	0.02	0.032	0.028	0.032
Overall	0.58 ^b^	0.60 ^ab^	0.63 ^a^	0.64 ^a^	0.03	0.011	0.028	0.013
Feed efficiency, kg/kg								
Pre-weaning	0.51	0.53	0.56	0.53	0.01	0.286	0.226	0.100
Post-weaning	0.47 ^b^	0.55 ^a^	0.55 ^a^	0.50 ^b^	0.02	0.031	0.034	0.025
Overall	0.50 ^b^	0.54 ^a^	0.55 ^a^	0.51 ^b^	0.01	0.012	0.032	0.017
Fecal score								
Pre-weaning	1.53 ^a^	1.45 ^ab^	1.42 ^b^	1.42 ^b^	0.03	0.039	0.008	0.125
Post-weaning	1.26	1.19	1.14	1.16	0.02	0.325	0.032	0.742
Overall	1.39	1.30	1.29	1.30	0.02	0.073	0.025	0.513

^a,b^ Mean values within a row with different superscripts differ significantly (*p* < 0.05). ^1^ Pre- and post-weaning: calves were given treatments from 28 to 50 d of age and from 56 to 80 d of age. Overall: calves were given treatments from 28 to 80 d of age. DMI = dry matter intake. Feed efficiency = daily gain/daily total DM intake. ^2^ CON = no yeast or flavonoids fed; ADY = *Candida tropicalis* as supplement; FLA = mulberry leaf flavonoids as supplement; YF = combination of *Candida tropicalis* and mulberry leaf flavonoids as supplement. ^3^ SEM = standard error of the mean.

**Table 3:** Effects of supplementation with *Candida tropicalis* and mulberry leaf flavonoids individually or in combination on rumen fermentation parameters in Holstein calves during pre-weaning, post-weaning, and overall periods.

Item ^1^	Treatment ^2^				SEM ^3^	*p*-Value		
	CON	ADY	FLA	YF		Treatment	Time	Treatment × Time
pH								
Pre-weaning	6.2	6.5	6.3	6.3	0.8	0.213	0.565	0.182
Post-weaning	6.2	6.4	6.4	6.3	0.7	0.397	0.020	0.191
Overall	6.1	6.4	6.3	6.3	0.7	0.265	0.154	0.290
NH_3_-N, mmol/L								
Pre-weaning	4.44	4.22	5.18	4.09	1.71	0.260	0.380	0.513
Post-weaning	7.52	7.36	7.40	7.33	1.05	0.340	0.280	0.316
Overall	5.49	5.42	5.47	5.18	1.92	0.213	0.342	0.253
Total VFA, mmol/L								
Pre-weaning	44.20 ^b^	49.99 ^b^	57.81 ^a^	60.90 ^a^	3.21	0.027	0.018	0.014
Post-weaning	75.96	81.45	71.27	83.85	5.27	0.253	0.561	0.316
Overall	59.19 ^b^	64.40 ^b^	66.83 ^ab^	74.12 ^a^	4.35	0.031	0.002	0.025
Acetate/total VFA, %								
Pre-weaning	57.60	61.40	61.17	56.67	3.35	0.917	0.041	0.635
Post-weaning	55.13	56.75	52.50	58.75	2.12	0.208	0.466	0.446
Overall	54.14	54.36	56.21	55.79	2.04	0.849	0.617	0.559
Propionate/total VFA, %								
Pre-weaning	24.20 ^b^	25.40 ^b^	29.50 ^ab^	32.83 ^a^	2.92	0.047	0.036	0.038
Post-weaning	24.09 ^b^	29.50 ^a^	30.13 ^a^	30.50 ^a^	1.64	0.045	0.747	0.838
Overall	24.13 ^b^	27.92 ^a^^b^	29.86 ^a^^b^	31.50 ^a^	1.84	0.018	0.026	0.017
Butyrate/total VFA, %								
Pre-weaning	6.40	8.60	6.83	7.00	1.16	0.455	0.108	0.740
Post-weaning	6.63	9.75	13.25	11.38	1.87	0.055	0.337	0.839
Overall	6.54	9.31	10.50	9.50	1.07	0.193	0.004	0.669
Acetate/propionate ratio								
Pre-weaning	2.37 ^a^	2.41 ^a^	2.05 ^ab^	1.77 ^b^	0.30	0.047	0.055	0.044
Post-weaning	2.29 ^a^	1.92 ^b^	1.74 ^b^	1.93 ^b^	0.19	0.031	0.618	0.779
Overall	2.24 ^a^	1.95 ^ab^	1.88 ^b^	1.77 ^b^	0.26	0.042	0.051	0.033

^a,b^ Mean values within a row with different superscripts differ significantly (*p* < 0.05). ^1^ Pre- and post-weaning: calves were given treatments at ages 28 and 42 d, and at ages 56 and 80 d. Overall: calves were given treatments at ages 28, 42, 56, and 80 d. ^2^ CON = no yeast or flavonoids fed; ADY = *Candida tropicalis* as supplement; FLA = mulberry leaf flavonoids as supplement; YF = combination of *Candida tropicalis* and mulberry leaf flavonoids as supplement; VFA = volatile fatty acids. ^3^ SEM = standard error of the mean.

**Table 4:** Effects of supplementation with *Candida tropicalis* and mulberry leaf flavonoids individually or in combination on blood parameters in Holstein calves during pre-weaning, post-weaning, and overall periods.

Item ^1^	Treatment ^2^				SEM ^3^	*p*-Value		
	CON	ADY	FLA	YF		Treatment	Time	Treatment × Time
IgG, g/L								
Pre-weaning	8.93 ^b^	9.12 ^b^	9.64 ^a^	9.68 ^a^	0.19	0.032	0.341	0.008
Post-weaning	9.45	9.62	9.74	10.15	0.27	0.120	0.258	0.364
Overall	9.09 ^b^	9.27 ^ab^	9.72 ^a^	9.78 ^a^	0.19	0.041	0.003	0.052
IgM, g/L								
Pre-weaning	0.84	0.83	0.83	0.86	0.01	0.438	0.204	0.511
Post-weaning	0.85	0.91	0.90	0.90	0.16	0.085	0.338	0.213
Overall	0.84	0.86	0.85	0.87	0.01	0.255	0.111	0.302
IgA, g/L								
Pre-weaning	1.96	1.93	1.92	2.05	0.02	0.551	0.399	0.612
Post-weaning	2.13 ^b^	2.07 ^b^	1.95 ^b^	2.40 ^a^	0.02	0.013	0.632	0.021
Overall	2.02 ^b^	1.98 ^b^	1.93 ^b^	2.20 ^a^	0.04	0.031	0.031	0.333
BHBA, mmol/L								
Pre-weaning	0.30	0.35	0.33	0.34	0.02	0.487	0.677	0.478
Post-weaning	0.43 ^b^	0.55 ^a^	0.56 ^a^	0.52 ^ab^	0.04	0.024	0.012	0.015
Overall	0.38	0.41	0.42	0.41	0.31	0.181	<0.001	0.406
EGF, pg/mL								
Pre-weaning	28.83 ^ab^	26.47 ^b^	35.38 ^a^	28.04 ^ab^	1.49	0.047	0.193	0.180
Post-weaning	30.23	28.29	33.44	29.57	2.07	0.217	0.418	0.321
Overall	29.44	27.23	34.89	28.94	1.80	0.161	0.586	0.606

^a,b^ Mean values within a row with different superscripts differ significantly (*p* < 0.05). ^1^ Pre- and post-weaning: calves were given treatments at ages 28 and 42 d, and at ages 56 and 80 d. Overall: calves were given treatments at ages 28, 42, 56, and 80 d. ^2^ CON = no yeast or flavonoids fed; ADY = *Candida tropicalis* as supplement; FLA = mulberry leaf flavonoids as supplement; YF = combination of *Candida tropicalis* and mulberry leaf flavonoids as supplement; BHBA = β-hydroxybutyric acid; EGF = epidermal growth factor. ^3^ SEM = standard error of the mean.
